# Mineral Paste for Filling Teeth

**Published:** 1844-06

**Authors:** S. M. Shepherd

**Affiliations:** Petersburg, Va.


### ARTICLE V.

### *Mineral Paste for Filling Teeth.*

MESSRS. EDITORS—I have recently seen several communications published, condemning the use of mineral paste in filling teeth. I am glad to see that some of the profession are determined to turn their faces against it. It has always been a matter of surprise to me, that any dentist should use it, especially when its component parts are known. I believe it is found by analysis, to be chiefly a compound of mercury and silver, no matter by what name it may be called. If it is true that these two things combined constitute "the mineral paste," no one need be surprised to find in some constitutions such malignant cases as were detailed by a member of the profession in the city of Baltimore, not long since. If the public generally could be made acquainted with a few such cases, it would require but little effort on the part of those who are resolved to discountenance its use, to put it down, and I know no better plan by which to do this, than to take a decided stand against it, in every shape and form.

I have never met with any one in my practice, who had had the paste used extensively, and I never saw a case where the result had proved so serious as in those above referred to; though I have met with many in which its injurious effects were pretty strongly marked. I presume that a person whose constitution is unimpaired by sickness, and who has no dormant mercurial medicines in the system, might have these paste fillings put in his teeth, without suffering such serious consequences, provided they were not very large or numerous. But if, on the other hand, the constitution has been injured, and the system impaired from the use of these mercurial medicines, I believe the introduction of these fillings could but produce a serious result. I do not know how much the digestive organs might be affected by the washings of such fillings, before it would be developed on the teeth and gums, or how much the whole system might suffer without giving a clue to the cause.

But suppose no actual injury could ever be traced to these fillings as the cause, still there remains an insuperable objection to their use. It is well known that all metals are subject to the laws of expansion and contraction; and although it requires but a slight heat to fuse the mineral paste, yet this heat must cause a slight expansion, and when this heat is expelled, a corresponding contraction must take place. It is well known, also, to dentists, that one important requisition in filling a tooth in such a manner as to arrest decay, is, that it must be so perfectly filled as to exclude even the moisture of the mouth; so that the slightest contraction of the filling, after it is put into the tooth, would render it of no avail.

But if any or all of the objections which I have urged above, can be invalidated by the advocates of the royal paste, still practice is better than theory. Facts will show that mineral paste is not the thing to fill teeth with. I have, in the course of my practice, removed a large number of these fillings, after they had remained in for some time, and to the best of my recollection, I never removed one under which I did not find the tooth in a rapid state of decay, and the final loss of the tooth would have been certain if it had not been refilled.

About a year ago, a young man who resides in the south, called on me to operate on his teeth, a large number of which were badly decayed, two of which I perceived had been *filled* with mineral paste. He told me it was done by a *celebrated* dentist in New Orleans, and he was unwilling to have the fillings removed, and stated that they gave him no trouble, and he supposed that they were doing well. But when I insisted on their removal, and assured him that he would lose the teeth if he did not consent to it, he consented, and I proceeded to remove them; and to his very great surprise I found about as much carious matter in the cavities as there was metal. Still the nerves of the teeth were not exposed. I ultimately filled these teeth permanently with gold, very much to the young man's satisfaction. I ought to remark, that in this case the two fillings adjoined each other, in the superior bicuspides of the right side, and that there was considerable inflammation in the gum. The inflammation very soon disappeared after the paste fillings were removed.

One other case I will mention, though I might mention many more: The Rev. Mr. ——, who resided in Philadelphia at the time the celebrated Mallan was in this country, had several of his teeth filled by that individual at that time, and kept the fillings in them until some time last fall, when he called on me and had one or two of them removed. One of the teeth, especially, had given him a good deal of trouble, and for some time he thought he would have the tooth removed. I removed the filling, however, and found much more carious matter under the filling than in the last case described. In this case the nerve had been destroyed, I suppose, by the process of decay under the filling. After removing the filling and a large portion of the carious matter, I let the cavity remain open for a few days, and the uneasiness gradually left it; I then filled the tooth thoroughly with gold, and I heard no more complaint from him about it.

In conclusion, I would only ask those who advocate the use of this paste in filling teeth, to remove one of these fillings—a large one—that has been in the tooth for one or two years; and if they do not become convinced that it is not the thing to fill teeth with, I think I might safely promise to yield the point.

S. M. Shepherd.

*Petersburg, Va. Feb.*, 1844.

